# Supplementary material for: HIVprotI: an integrated web based platform for prediction and design of HIV proteins inhibitors
Source: J Cheminform. 2018 Mar 9;10:12. doi: 10.1186/s13321-018-0266-y (PMC5845081; doi:10.1186/s13321-018-0266-y)
Supplement: Supplementary file 2 — Additional file 2. Source code of HIVProtI web server. [file 13321_2018_266_MOESM2_ESM.zip › HIVprotI_Source-code/hivprotI/weka-3-6-6/doc/weka/experiment/PairedStatsCorrected.html]

PairedStatsCorrected


---


|  |  |  |
| --- | --- | --- |
| **Overview** \| **Package** \| **Class** \| **Tree** \| **Deprecated** \| **Index** \| **Help** \| **Weka's home** | |  |
| **PREV CLASS**   **NEXT CLASS** | **FRAMES**    **NO FRAMES**     **All Classes** |  |
| SUMMARY: NESTED \| FIELD \| CONSTR \| METHOD | DETAIL: FIELD \| CONSTR \| METHOD |  |


---


## weka.experiment Class PairedStatsCorrected

```
java.lang.Object
  weka.experiment.PairedStats
      weka.experiment.PairedStatsCorrected
```

**All Implemented Interfaces:**: RevisionHandler

---

``` public class PairedStatsCorrected extends PairedStats ```

A class for storing stats on a paired comparison. This version is
based on the corrected resampled t-test statistic, which uses the
ratio of the number of test examples/the number of training examples.

For more information see:

Claude Nadeau and Yoshua Bengio, "Inference for the Generalization Error,"
Machine Learning, 2001.

**Version:**
:   $Revision: 1.5 $

**Author:**
:   Richard Kirkby (rkirkby@cs.waikato.ac.nz)

---

**Field Summary**

**Fields inherited from class weka.experiment.PairedStats**

`correlation, count, differencesProbability, differencesSignificance, differencesStats, sigLevel, xStats, xySum, yStats`


**Constructor Summary**

`PairedStatsCorrected(double sig,
double testTrainRatio)`
  
          Creates a new PairedStatsCorrected object with the supplied
significance level and train/test ratio.


| **Method Summary** | |
| --- | --- |
| `void` | `calculateDerived()`             Calculates the derived statistics (significance etc). |
| `java.lang.String` | `getRevision()`             Returns the revision string. |

**Methods inherited from class weka.experiment.PairedStats**

`add, add, getDegreesOfFreedom, main, setDegreesOfFreedom, subtract, subtract, toString`

**Methods inherited from class java.lang.Object**

`equals, getClass, hashCode, notify, notifyAll, wait, wait, wait`

**Constructor Detail**

### PairedStatsCorrected

```
public PairedStatsCorrected(double sig,
                            double testTrainRatio)
```

:   Creates a new PairedStatsCorrected object with the supplied
    significance level and train/test ratio.

    **Parameters:**: `sig` - the significance level for comparisons: `testTrainRatio` - the number test examples/training examples


**Method Detail**

### calculateDerived

```
public void calculateDerived()
```

:   Calculates the derived statistics (significance etc).

    :   **Overrides:**: `calculateDerived` in class `PairedStats`

---


### getRevision

```
public java.lang.String getRevision()
```

:   Returns the revision string.

    :   **Specified by:**: `getRevision` in interface `RevisionHandler` **Overrides:**: `getRevision` in class `PairedStats`
    :   **Returns:**: the revision


---


|  |  |  |
| --- | --- | --- |
| **Overview** \| **Package** \| **Class** \| **Tree** \| **Deprecated** \| **Index** \| **Help** \| **Weka's home** | |  |
| **PREV CLASS**   **NEXT CLASS** | **FRAMES**    **NO FRAMES**     **All Classes** |  |
| SUMMARY: NESTED \| FIELD \| CONSTR \| METHOD | DETAIL: FIELD \| CONSTR \| METHOD |  |


---
